# Wuzi-Yanzong prescription alleviates spermatogenesis disorder induced by heat stress dependent on Akt, NF-κB signaling pathway

**DOI:** 10.1038/s41598-021-98036-2

**Published:** 2021-09-22

**Authors:** Su-Qin Hu, Dian-Long Liu, Chun-Rui Li, Ya-Hui Xu, Ke Hu, Li-Dan Cui, Jian Guo

**Affiliations:** Department of Physiology, College of Traditional Chinese Medicine, Beijing University of Traditional Chinese Medicine, No. 11, East Beisanhuan Road, Chaoyang District, Beijing, China

**Keywords:** Molecular biology, Endocrinology, Medical research, Pathogenesis

## Abstract

Akt and nuclear factor kappa B (NF-κB) signaling pathways are involved in germ cell apoptosis and inflammation after testicular heat stress (THS). We observed that after THS induced by the exposure of rat testes to 43 °C for 20 min, their weight decreased, the fraction of apoptotic testicular germ cells significantly increased, and the proliferation of germ cells was inhibited. In addition, THS lowered serum testosterone (T) level, whereas the levels of follicle stimulating hormone and luteinizing hormone were not significantly changed. The ultrastructure of the seminiferous tubules became abnormal after THS, the structure of the blood-testis barrier (BTB) became loose, and the Sertoli cells showed a trend of differentiation. The level of phosphorylated Akt was reduced, whereas the amount of phosphorylated NF-κB p65 was augmented by THS. Wuzi-Yanzong (WZYZ), a classic Chinese medicine prescription for the treatment of male reproductive dysfunctions, alleviated the changes induced by THS. In order to determine the mechanism of action of WZYZ, we investigated how this preparation modulated the levels of T, androgen receptor (AR), erythropoietin (EPO), EPO receptor, and Tyro-3, Axl, and Mer (TAM) family of tyrosine kinase receptors. We found that WZYZ activated the Akt pathway, inhibited the Toll-like receptor/MyD88/NF-κB pathway, and repaired the structure of BTB by regulating the levels of T, AR, TAM receptors, and EPO. In conclusion, these results suggest that WZYZ activates the Akt pathway and inhibits the NF-κB pathway by acting on the upstream regulators, thereby improving spermatogenesis deficit induced by THS.

## Introduction

Testicular heat stress (THS) refers to the stress response of the testis in a high-temperature environment^1^. For most mammals, the testes drop into the scrotum before or shortly after the early neonatal period, and the temperature of the scrotum is generally 2–7 °C lower than the core body temperature^2^. Spermatogenesis is more sensitive to the temperature, and high temperature around the scrotum can lead to the apoptosis of heat-sensitive testicular germ cells^3^, THS may arise as a result of occupational exposure to high temperature and affect spermatogenesis^4^. In daily life, an increasing number of men are exposed to short-term or long-term high-temperature conditions due to their occupations (e.g., sedentary desk workers, welders, drivers, construction workers, cement production workers, and steel workers^5–7^). It has been shown that THS not only induces germ cell apoptosis^8^, but also causes an inflammatory reaction^9^ that aggravates testicular injury. Apoptosis and inflammation in the testis are mainly regulated by the Akt and NF-κB signaling systems^10–12^. Therefore, we speculated that the NF-κB and Akt signaling pathways could be deeply involved in testicular inflammation and germ cell apoptosis after THS.

Defining how Akt and NF-κB participate in THS requires dissection of the Akt pathway, the activation of which prevents germ cell death. Akt is largely implicated in cell cycle regulation, apoptosis inhibition, and cell proliferation^13^. Akt knockout animals showed increased apoptosis in several tissue types^14^, as well as defects in growth and development^15^, Akt has also been demonstrated to inhibit germ cell apoptosis after testicular X-ray irradiation in adult mice^11^. Nuclear factor kappa B (NF-κB) protein complexes consisting of five NF-κB family proteins are one of the most important mediators of the inflammatory response^16^. They exist as homodimers and heterodimers, and the most characteristic form is the heterodimer comprising the p65 subunit^17^. Toll-like receptors (TLRs), a family of pattern recognition receptors, are the first line of defense of the testis against bacterial and viral pathogens^18^. In addition, it has been reported that TLRs activate the initial inflammatory signal during testicular tissue injury^19–22^, TLRs were initially thought to recognize only specific bacteria, and they have been shown later to be activated by many signals induced by stress or cell injury^23^. Common TLR signaling is mediated through an adaptor protein, MyD88, which recruits other intermediate molecules that in turn activate p-IκBα. Futhermore, this degradation liberates p-NF-κB for translocation into the nucleus, where it directs transcription of the TLR response cytokines such as interleukin (IL)-1β, IL-2, IL-4, and tumor necrosis factor α (TNF-α) are induced^24,25^. Some studies have shown that inhibition of the NF-κB signaling pathway can protect against testicular injury induced by THS^26^, but the mechanism of this effect has not been further explained.

The androgen testosterone (T) plays an important role in maintaining spermatogenesis and male sexual characteristics^27^. T activates Akt signaling through the androgen receptor (AR) pathway, and plays an important role in regulating cell proliferation and apoptosis^28–30^. In addition, there is evidence that T inhibits the NF-κB signaling pathway and downregulates the expression of TNF-α in endothelial cells^31^. It has also been reported that T attenuates NF-κB activation and decreases the expression of IL-1 and TNF-α in prostate smooth muscle cells by downregulating TLR4^32^. Erythropoietin (EPO) is a heat-resistant glycoprotein that has been shown to play an anti-apoptotic role by activating Akt^33^. In addition, it has been reported that EPO plays an anti-inflammatory role in acute renal inflammation induced by crush syndrome^34^ and in the inflammation induced by lung ischemia/reperfusion^35^ by inhibiting the TLR4/NF-κB signaling pathway. Tyro-3, Axl, and Mer (TAM) family of tyrosine kinase receptors is an important testicular immune regulatory factor^36,37^, TAM receptors have been shown to negatively regulate the TLR/NF-κB signaling pathway. However, whether T, EPO and TAM receptors play roles in the THS-induced spermatogenesis dysregulation remains unclear.

Pharmacological treatments (e.g., gonadotropin replacement therapy, dopamine receptor agonists, and antioxidant supplements) have been used to treat spermatogenesis disorders, but none of such treatments has a satisfactory effect on the spermatogenesis dysregulation induced by THS^38^. People have been concerned about the use of complementary and alternative drugs. In recent years, several traditional Chinese medicine prescriptions for the treatment of male reproductive disorders have been used to reverse testicular tissue damage caused by THS^39^. The Wuzi-Yanzong pill (WZYZ) composed of five plants (Table 1) is a classical Chinese medicine prescription thought to stimulate the kidney and promote male fertility; it has been first mentioned in the medical records of the Tang Dynasty of China. Previous studies have shown that WZYZ improved the quality of testicular spermatozoa in rats with oligozoospermia induced by multiglycosides of *Tripterygium wilfordii*^40^, alleviated testicular damage induced by ionizing radiation^41^, and promoted spermatogenesis by regulating the secretory function of Sertoli cells (SCs) and upregulating T levels^42^.

**Table 1:** Composition of Wuzi-Yanzong prescription(WZYZ).

Botanical name	Chinese name	Lot number	Part used	Proportion (%)
*Lycium barbarum* L	Gouqizi	19014101	Fruit	33.3
*Cuscuta chinensis* Lam	Tusizi	19002221	Fruit	33.3
*Rubus chingii* Hu	Fupenzi	19019791	Fruit	16.7
*Schizandra chinensis* (Turcz.) Baill	Wuweizi	19016171	Fruit	8.3
*Plantago asiatica* L	Cheqianzi	19034531	Fruit	8.3

In this study, the widely accepted testicular acute THS model^43^ was used to investigate whether WZYZ can reverse THS-induced spermatogenesis deficit via Akt and NF-κB signaling pathways through the modulation of T, EPO, and TAM receptors.

## Materials and methods

### Animals

The male Wistar rats aged 60-day were purchased from Beijing Vital River Laboratories. Ltd. (Beijing, China). Animals were maintained in a standard animal facility under temperature and humidity-controlled room on a 12 h light/dark cycle, with free access to water and standard rat chow diet. They were treated and sacrificed according to the ARRIVE guidelines (PLoS bio 8 (6), e1000412, 2010). All animals were under the guidelines of the Laboratory Animal Welfare and Ethics Committee of Beijing University of Chinese Medicine (BUCM-4-2019030701-1053).

### Preparation of WZYZ and chromatogram analysis

**Figure 1:**
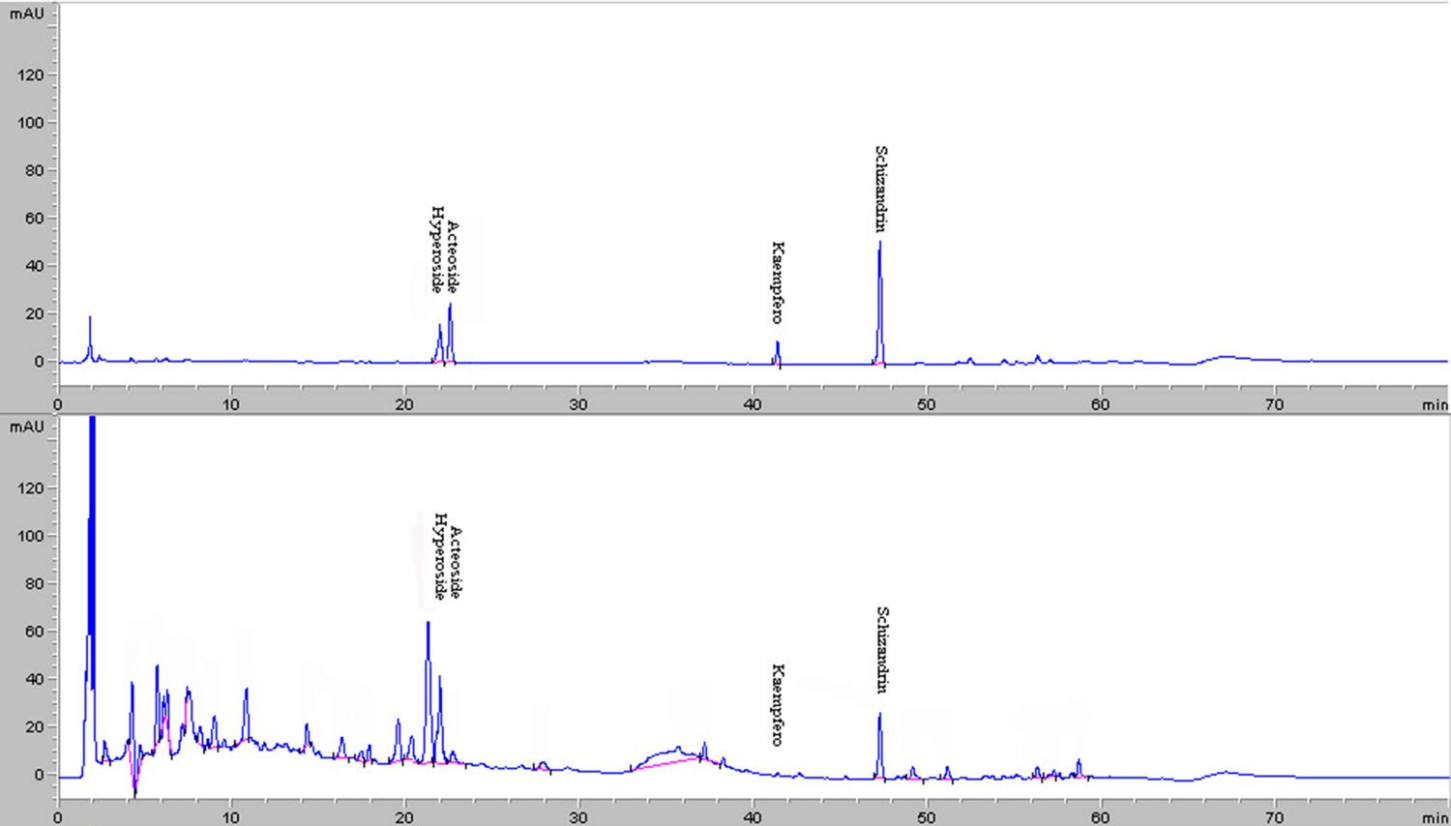
Control characteristic map (chromatogram) of WZYZ.

WZYZ was provided by the Beijing kangrentang Pharmaceutical Co., Ltd (Beijing, China), included the five components (Table 1), each herbal medicine contains a variety of ingredients in different amounts. According to the Chinese Pharmacopoeia, there should be 5 characteristic peaks in the specific chromatogram, and the technical regulations for hyperin (C_21_H_20_O_12_) and schisandrin (C_24_H_32_O_7_) were made, that is, hyperoside should not be less than 0.20 mg per 1 g water-honeyed pills, and schisandrin should not be less than 0.10 mg per 1 g water-honeyed pills. In this study, we used High-Performance Liquid Chromatography (HPLC) to analyze the components of hyperin (C_21_H_20_O_12_) and schisandrin (C_24_H_32_O_7_). The chromatographic column was C18 column (4.6 × 150 mm, 5 μm) and the chromatographic separation conditions were as follows: Column temperature: 30 °C; Flow rate: 1.0 ml/min; Filler: octadecylsilane bonded silica gel; Mobile phase: acetonitrile-methanol (10:1) + 0.4% phosphoric acid (0–10 min, 5%A → 15%A; 10–20 min, 15%A → 19%A; 25–30 min, 19%A → 21%A; 20–30 min, 19%A → 21%A; 30–60 min, 21%A → 90%A; 60–65 min, 90%A → 5%A;); Detection wavelength: 360 nm for hyperin, 250 nm for schisandrin; Stock solutions of WZYZ was prepared by dissolving 3 g of analyte in 25 ml of 70% methanol. Content in Wuzi-Yanzong prescription was determined by quantitation hyperin (C_21_H_20_O_12_) and schisandrin (C_24_H_32_O_7_), The characteristic map (chromatogram) of Wuzi-Yanzong prescription(WZYZ) is shown in (Fig. 1). The content of hyperin and schisandrin is shown in (Table 2). WZYZ was dissolved with physiological saline (Shijiazhuang No.4 Pharmaceutical, Hebei, China) to prepare for use.

**Table 2:** The content of hyperin and schisandrin in WZYZ.

Compound name	Determination wavelength (nm)	R_T_ (t_R_, min)	Amount (mg/g)
Hyperin	360	21.88	0.81
Schisandrin	250	47.21	0.25

### Regents

Rabbit anti-Ki67 antibody (Cat. No. ab16667), Rabbit anti-Cytokeratin 18 antibodies (Cat. No. ab16667), and Rabbit anti-Androgen Receptor antibody (Cat. No. ab16667) were purchased from Abcam (Cambridge, UK). Rabbit anti-Phospho-Akt (Ser473) (Cat. No. #4060), anti-Akt (Cat. No. #4691), anti-NF-κB p65(Cat. No. #8242), anti-Phospho-NF-κB p65(Cat. No. #3033), anti-IκBα (Cat. No. #4812), anti-Phospho-IκBα (Cat. No. #2859) were obtained from Cell Signaling Technology (Boston, MA, USA). Rabbit anti-Erythropoietin R (Cat. No. NBP1-19388) was purchased from Novus Biologicals (Littleton, CO, USA). And rabbit anti-Erythropoietin (Cat. No. LS-B15015) was purchased from LifeSpan BioSciences (Seattle, WA, USA). The anti-beta-Actin (Cat. No. bs-10966R) was obtained from Beijing Biosynthesis Biotechnology CO., LTD (Beijing, China).

### Experimental protocol

The 40 rats were randomly divided into five groups (n = 8 rat per group) : control, model, low-dose WZYZ, medium-dose WZYZ, and high-dose WZYZ groups. Rats in the WZYZ groups were pretreatment with 418 mg/kg/d, 836 mg/kg/d, and 1672 mg/kg/d, rats in control and model groups were given an equivalent volume of physiological saline, respectively, by gavage twice daily for 15 days. After anesthesia with an intraperitoneal injection of sodium pentobarbital (40 mg/kg body weight), the tails and the scrotum containing testes were then immersed in a thermostatically controlled water bath at 43 °C for 20 min. Control rats were treated in the same way, except the testes were immersed in a water bath maintained at 22 °C. Then we sacrificed these animals 30 min after heat stress.

### Hormone level in serum

The levels of Follicle-stimulating hormone (FSH), Luteinizing hormone (LH), and Testosterone (T) in serum were measured by radioimmunoassay (RIA), as reported previously^44^.

### Determination of serum inflammatory factors

For the measurement of inflammatory factors in rat serum, fresh abdominal aortic blood was collected and centrifuged at 3000×*g* for 10 min. The serum was carefully collected and frozen to − 80 °C. The IL-1b, IL-2, IL-4, and TNF-α levels were measured using Rat Inflammation Array Q1(QAR-INF-1-2, RayBiotech, Guangzhou, China) according to the manufacturer’s instructions.

### Evaluation of cell apoptosis

Germ cell apoptosis was evaluated by the terminal deoxynucleotidyl transferase dUTP nick end labeling (TUNEL) assay. After testis fixing in Modified Davidson’s Fluid and embedding in paraffin, the paraffin mass was sliced into 4–5 μm paraffin sections. Paraffin section of testis tissue was dewaxed, hydrated with gradient alcohol, and washed. And TUNEL staining was examined by the TUNEL detection kit-POD (Roche, Basel, Switzerland). Images were captured from a microscope (BX53; Olympus Optical). ImageJ Version 1.6.0_20 (National Institutes of Health, USA) was utilized to analyze the TUNEL-positive area.

### Immunohistochemistry

Immunohistochemistry was performed as described previously^44^. The processes could be described as follows: paraffin sections of testis tissue were dewaxed by washing three times for 30 min each in xylene, then rehydrated in 100%, 95%, 80%, and 70% ethanol, for 5 min each, before finally being rinsed with distilled water. The sections were washed and treated with 3% hydrogen peroxide in methanol for 10 min in order to inhibit the activity of any endogenous peroxide. After three times washing with 0.05 M phospate-buffer saline (PBS), the sections were in citrate buffer and treated in a microwave oven using 750 Watt power for 15 min (3 × 5 min). After complete cooling, the sections were treated with rabbit or goat serum for 30 min at room temperature to block nonspecific protein staining. The sections were incubated with Anti-Ki67 antibody (1:100) at 4 °C overnight. After three times washing, the sections were treated with Polymer Helper for 20 min at 37 °C. After three times washing, poly-HRP anti-Rabbit IgG was incubated for 20 min at 37 °C. After three times washing, to develop peroxidase activity, 3–3′diaminobenzidine tetrahydrochloride was used. After three times washing, the sections were subsequently counterstained with hematoxylin and were dehydrated, cleared, mounted. Images were captured from a microscope (BX53; Olympus Optical).

### Ultrastructural analysis by electron microscopy

Testicular tissues were fixed in 2.5% glutaric acid solution at 4 °C overnight and washed in 0.1 M PBS. The tissues were postfixed with 1% osmium tetraoxide for 2 h at room temperature before washing. Then they were dehydrated by graded acetone. Dehydrated tissues were processed for making Araldite blocks. Ultrathin sections were obtained by ultramicrotome (EMUC7; Leica) and collected on copper grids for double staining (uranyl acetate and Reynold’s lead citrate). Stained sections were finally observed under a HITACHI, H-7650 transmission electron microscope.

### RT-PCR analysis

Total RNA was extracted using the HiPure Total RNA Mini Kit (Magen BioTECH, Guangzhou, China) following the manufacturer’s instructions. RT–PCR was performed as described before^44^. The processes could be described as follows: the RNA(2 μg) was reverse transcribed into cDNA in 20 μl of the reverse transcriptase reaction mixture. For RT-PCR analysis, 2 μl of the cDNA was mixed with 1 μl of forward and reverse primers and 10 μl of 2 × master mix (Power SYBR Green PCR master mix; Applied Biosystems) to a final volume of 20 μl. Reactions were performed on a Bio-Rad CFX Maestro 1.0 ABI PRISM 7300 real-time cycler (Applied Biosystems, Foster City, CA) with a melting step of 95 °C for 10 min and a hot start step followed by 49 amplification cycles (95 °C, 10 s; 55 °C, 30 s; 65 °C, 5 s). Finally, the target cDNA was obtained by cooling to 4 °C and stored at − 20 °C. The primers used for Q-PCR are listed in (Table 3).

**Table 3:** List of primers used for RT-PCR.

Target genes	Primer pairs (5′ → 3′)	
	Forward	Reverse
TYRO-3	ACAACGATCTCCAGCAGCAACG	CACGGGAACCACGACAGCAAG
AXL	AACCCGTGACCCTGCTCTGG	GGTGGTGACTCCCTTGGCATTG
MERTK	AGTGAGCCAGTGGACGTAGCC	GCAGAAGCAGCCCAGGATGATG
GAS-6	TCAGGGCTGGGCGACTTGAG	ACATGCCGTGGTTGATGGTTGG
PROS-1	GCCCTGGTGCTCCCTGTCTC	AGGTATTTGCACGGCGCTTCC
TLR-4	ACTTTATCCAGAGCCGTTGGTGTATC	TCAAGGACAATGAAGATGATGCCAGAG
MyD-88	CAACCAGCAGAAACAGGAGTC	ATTGGGGCAGTAGCAGATGAAG

### Western blotting

Testicular tissues were homogenized in protein extraction buffer followed by centrifugation at 12,000×*g* for 10 min at 4 °C, and the supernatant was collected. Western Blotting was performed as described before^44^. The processes could be described as follows: Protein concentrations were determined by bicinchoninic acid protein assay. Samples (20 mg/lane) were separated by SDS–polyacrylamide gel electrophoresis and transferred to polyvinylidene difluoride membranes. The membranes were blocked with 5% (w/v) non-fat dry milk in Tris-buffered saline containing 0.1% Tween 20 (TBST) and were incubated with the following antibodies in TBST containing 5% non-fat dry milk at 4 °C overnight: Anti-beta-Actin (1:4000), Anti-Androgen Receptor (1:1000), Anti-Anti-Cytokeratin 18 (1:1000), Anti-Akt (1:1000), Anti-Phospho-Akt(1:1000), Anti-NF-κB p65(1:1000), Anti-Phospho-NF-κB p65 (1:1000), Anti-IκBα (1:1000), Anti-Phospho-IκBα (1:1000), Anti-Erythropoietin R Antibody (1:1000), Anti-Erythropoietin(1:500). After three times washing, the membranes were incubated with Horseradish peroxidase (HRP)-conjugated anti-rabbit immunoglobulin G (1:4000) (Beijing Biosynthesis Biotechnology CO, LTD, Beijing, China) for 1 h at room temperature. Band intensities were detected and analyzed by Quantity One software, Version 4.6.2 (Bio-Rad Laboratories, Hercules, CA, USA).

### Statistical analysis

All values are expressed as mean ± standard error of mean (SEM). Data in different groups were determined by one-way analysis of variance (ANOVA) and nonparametric test (Kruskal–Wallis) using SPSS statistical software (version 21.0; International Business Machine, New York, USA). A p value < 0.05 was considered statistically significant.

### Ethical approval

All animal care, husbandry and experimentation were performed according to the guidelines set by the Laboratory Animal Welfare and Ethics Committee of Beijing University of Chinese Medicine. All animal studies were approved by the Laboratory Animal Welfare and Ethics Committee of Beijing University of Chinese Medicine (BUCM-4-2019030701-1053). This study complies with the relevant specifications of the guide to advance (PLoS bio 8 (6), e1000412, 2010).

## Results

### WZYZ restores testicular weight and testosterone level in acuteTHS rats

**Figure 2:**
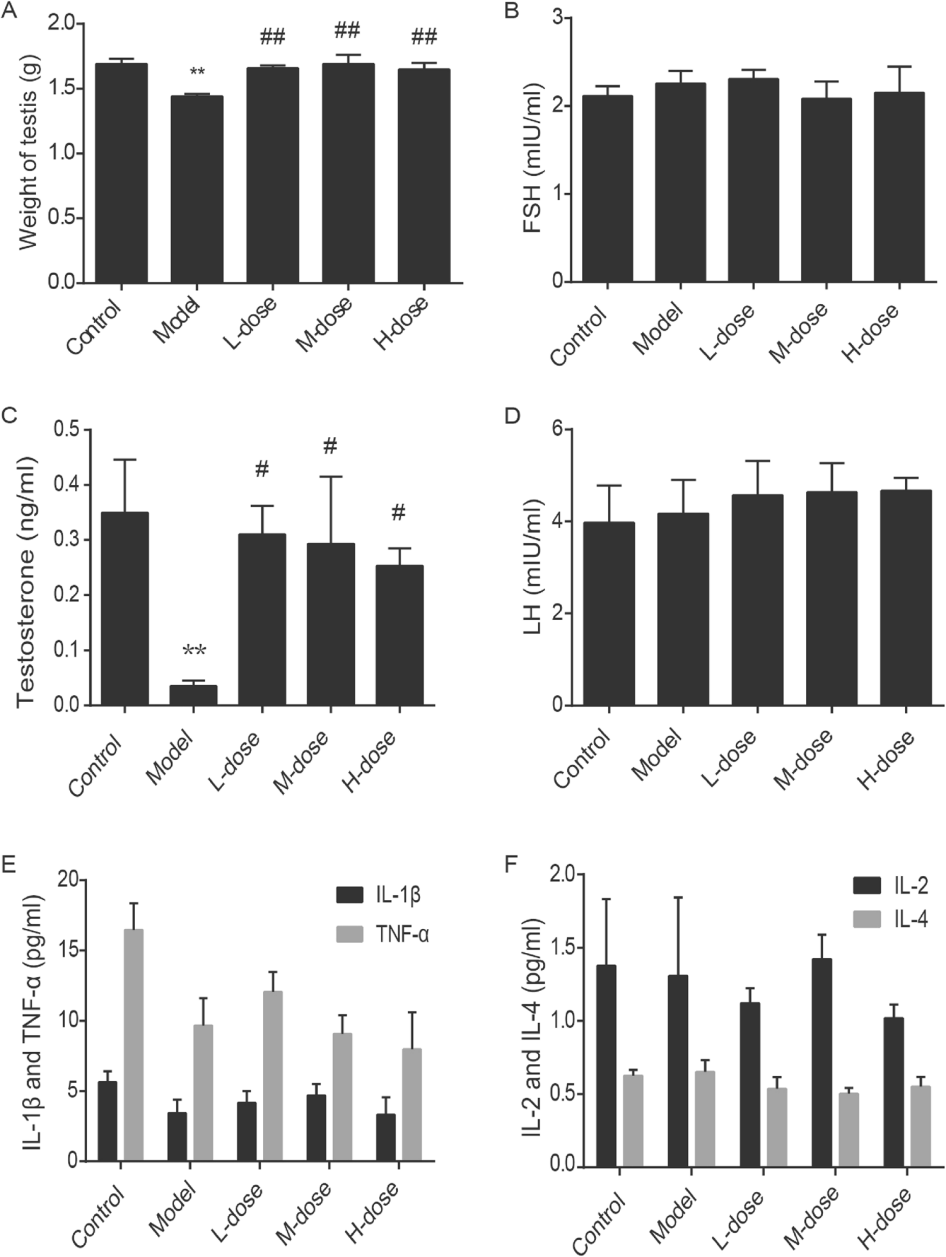
Pretreatment with gradient concentration WZYZ increased testicular weight and testosterone level in rats induced by acute THS. The weight of testis (**A**) and subsequent sex hormone levels, FSH (**B**), LH (**C**), testosterone (**D**), Inflammatory factors levels, IL-1β, TNF-α (**E**), IL-2 and IL-4 (**F**) were measured. The data showed an average ± SEM (n = 7 rat per group). *Compared with the control group, P < 0.05, **P < 0.01; compared with the model group, P < 0.05, P < 0.01.

We first measured testicular weight (Fig. 2A) and tested serum sex hormone (Testosterone, FSH, LH) levels by radioimmunoassay (Fig. 2B–D). After heat exposure for 20 min, the testicular weight and testosterone level of rats in the model group were significantly decreased, compared with the control group. And WZYZ treatment could increase the testicular weight and up-regulate Testosterone level compared with the model group, meanwhile different concentrations of WZYZ pretreatment can restore this trend, but there is no significant difference between different concentrations of WZYZ pretreatment groups. There was no significant difference in serum levels of FSH, LH, IL-1β, IL-2, IL-4 and TNF-α among groups.

### WZYZ reduced the apoptosis of germ cell in acute THS-induced rat testis

**Figure 3:**
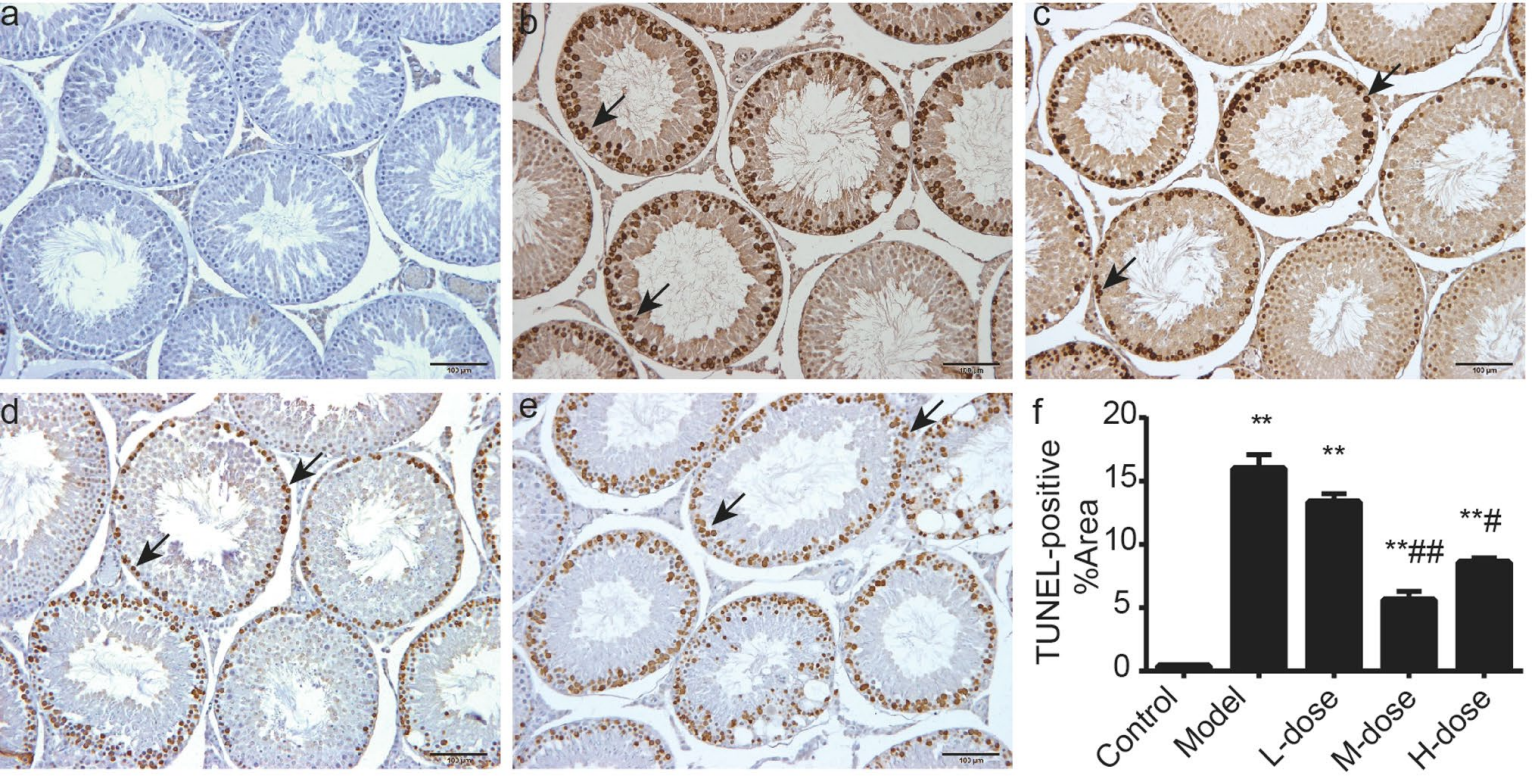
Pretreatment with a gradient concentration of WZYZ could reduce the apoptosis of germ cells induced by acute THS. Germ cell apoptosis was detected by TUNEL. The typical image shows that the brown area is TUNEL positive germ cells (black arrow). Bar = 100 μM. (**a**) Control group; (**b**) Model group; (**c**) Low-dose WZYZ group; (**d**) Middle-dose WZYZ group; (**e**) High-dose WZYZ group; (**f**) bar chart showing percentage of TUNEL positive area. The data showed an average ± SEM (n = 5 rat per group). **Compared with the control group, P < 0.01; compared with the model group, P < 0.05, P < 0.01.

The apoptosis of germ cells was assessed by the TUNEL assay (Fig. 3). In the control group, TUNEL-positive germ cell areas (mainly were spermatocytes) were barely displayed in seminiferous tubules. The TUNEL-positive germ cell area showed an obvious increase in the model group (p < 0.01). However, the TUNEL reactivity was decreased in WZYZ groups when compared with the model group (p < 0.05 or p < 0.01).

### WZYZ recovered proliferative viability of germ cell in acute THS-induced rat testis

**Figure 4:**
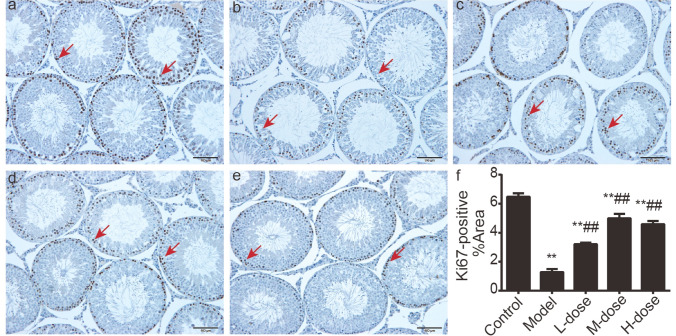
Pretreatment with a gradient concentration of WZYZ could restore the proliferation activity of rat testicular germ cells induced by acute THS. The expression of Ki67 in germ cells was detected by immunohistochemistry. A representative image shows Ki67 positive germ cells in the brown area (red arrow). Bar = 100 μM. (**a**) Control group; (**b**) model group; (**c**) low-dose WZYZ group; (**d**) middle-dose WZYZ group; (e) High-dose WZYZ group; (f) bar chart describing the percentage of Ki67 positive area. The data showed an average ± SEM (n = 3 rat per group). **Compared with the control group, P < 0.01; ##compared with the model group, P < 0.01.

To determine whether WZYZ could recover the expression of cell proliferation after scrotal hyperthermia, we evaluated the Ki67 in germ cells (Fig. 4). Ki67-positive signals were strongly detected in the germ cells (mainly were spermatogonia and spermatocytes) in the control group (Fig. 4a). A decrease of Ki67-positive germ cells was evident after mild testicular hyperthermia (Fig. 4b) when compared with the control group (p < 0.01). However, compared with the model group, Ki67-positive germ cells were significantly increased in seminiferous tubules after WZYZ treatment (p < 0.01).

### WZYZ ameliorated abnormal morphological structure of tissue and cell in THS-induced rat testis

**Figure 5:**
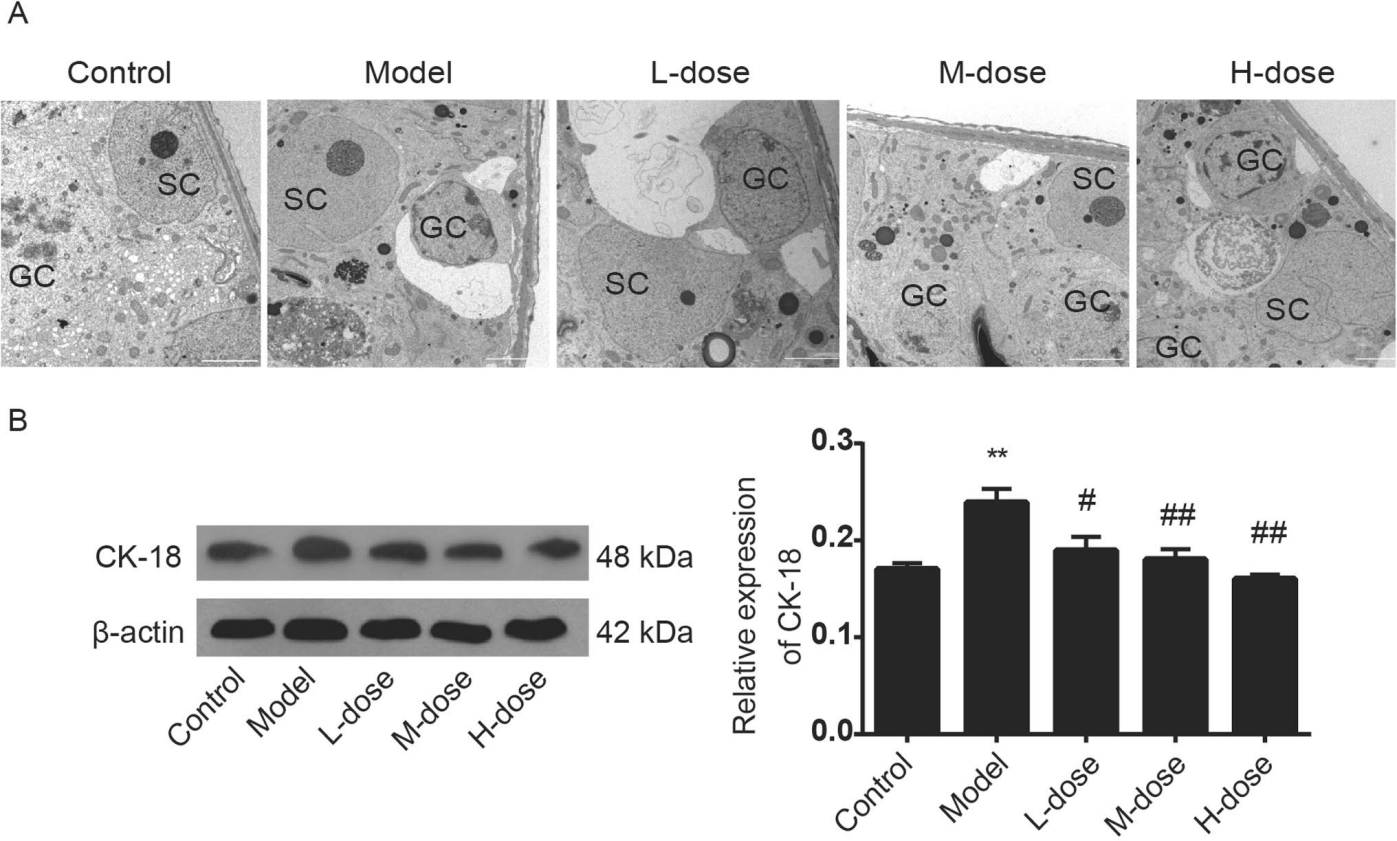
Pretreatment with a gradient concentration of WZYZ ameliorated abnormal morphological structure of tissue and cell in acute THS-induced rat testis. (**A**) Representative images of the ultrastructure in rat testis, bar: 5 microns. (**B**) Analysis of CK-18 expression in rat testis by Western blotting. Data are shown as mean ± SEM (n = 3 rat per group). **p < 0.01 compared with the control group; ^#^p < 0.05, ^##^p < 0.01 compared with the model group.

To investigate the effect of WZYZ on the structure of seminiferous tubules after heat exposure, we examined the ultrastructural changes of seminiferous tubules (Fig. 5A). SCs play an important role in mammalian spermatogenesis. Cytokeratin 18 (CK18) is a cytoskeleton molecule and is only expressed in immature SCs with the ability of mitosis and proliferation^45,46^. CK18 was detected in SCs of adult rat testis, indicating failure of maturation or reversal of maturation induced by environmental factors^47–49^. As shown by electron microscopic observation, in the control group, the morphology and structure of seminiferous tubules were complete, and the cell junctions (blood-testis barrier) were intact and compact. In the model group, the structure of seminiferous tubules became loose because of large vacuoles and wide intercellular spaces after heat exposure. In WZYZ treatment group, the arrangement of seminiferous tubules became slightly compact, but the whole structure was still very loose and could not return to normal.

The expression level of cytokeratin-18 was used to evaluate the maturity of SCs. As shown in Fig. 5B, the expression of CK-18 was rarely detected in the control group. After acute HS, the expression of CK-18 increased significantly compared with the control group (p < 0.01). However, the expression of CK-18 in WZYZ groups was decreased significantly compared with the model group (p < 0.01).

### WZYZ increased the expression of AR, p-Akt(Ser473),EPO and EPOR after THS

**Figure 6:**
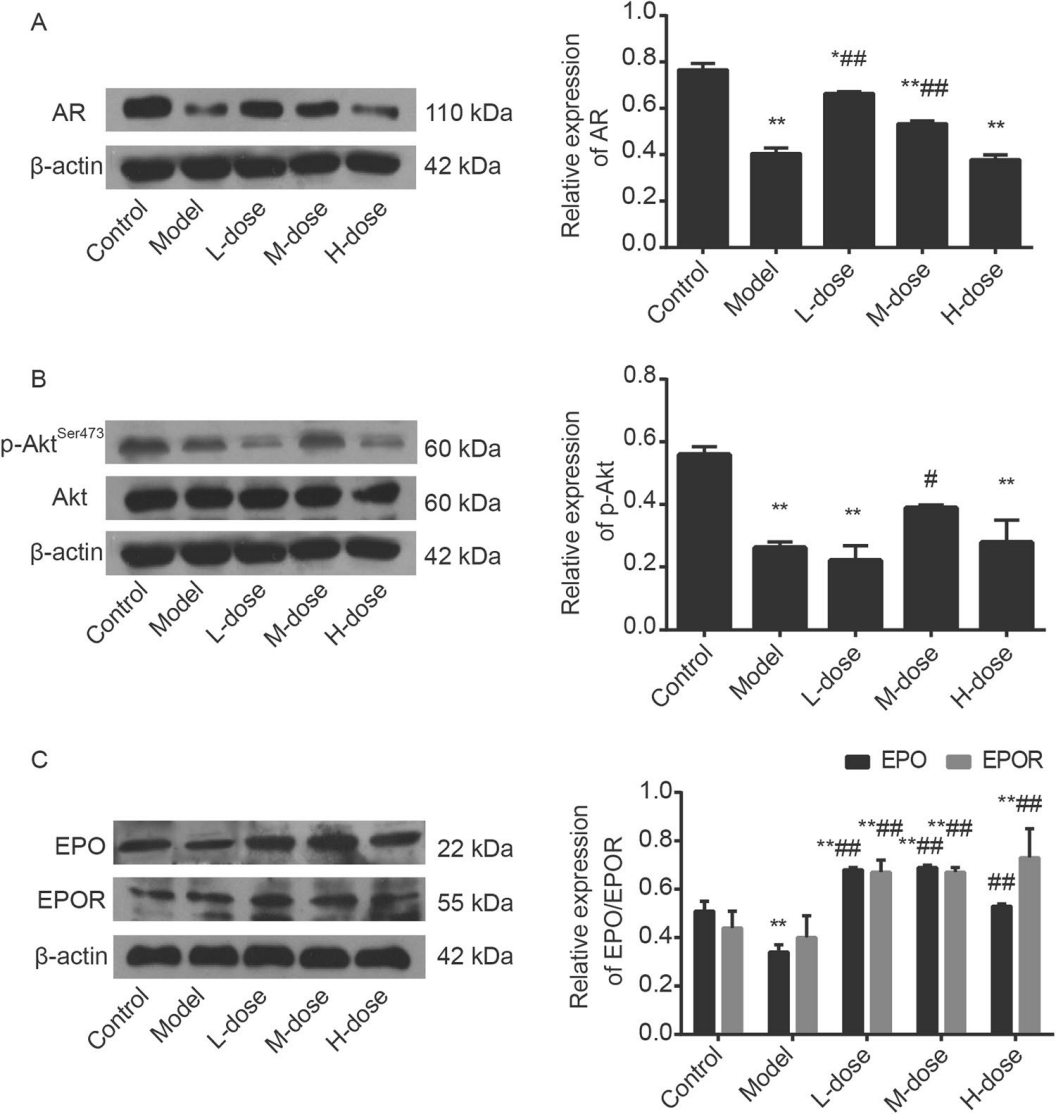
Pretreatment with a gradient concentration of WZYZ upregulated the expression of AR, p-Akt (Ser473), EPO, and EPOR in acute THS induced rat testis. Analysis of AR (**A**), p-Akt (Ser473) (**B**), EPO, and EPOR (**C**) expression in rat testis by Western blotting. Data are shown as mean ± SEM (n = 3 rat per group). *p < 0.05, **p < 0.01 compared with the control group; ^#^p < 0.05, ^##^p < 0.01 compared with the model group.

To look for possible regulation and mechanism of WZYZ underlying resist THS-induced impairment in testis, we further investigated changes in expression of AR, p-Akt (Ser473), EPO, and EPOR (Fig. 6). The representative results of Western blot analysis for AR was shown in Fig. 6A. T is the major androgen in the testes that are apparently required to support spermatogenesis. AR, which is mainly expressed in SCs of the testis, also plays an important role in the regulation of testicular cell junction and is essential for spermatogenesis^50,51^. It is the only specific receptor for androgen, the T diffuses into SCs binds to the AR to maintain spermatogenesis due to no functional receptors for androgen in germ cells^52,53^. The final expression of AR in SCs before maturation is considered to be a feature of the first mature SCs^54^. T and AR are important for Sertoli cell differentiation and regulating normal spermatogenesis^27,55–59^. Earlier studies have shown that the expression of Sertoli cell-specific AR affected SCs differentiation and maturation^27,57^, AR also involved in the expression of junction related molecules after THS^60^. The binding of AR with related ligands can increase the phosphorylation level of Akt^61^. The expression of AR in the Model decreased significantly when compared with the control group. WZYZ at Low-dose and Medium-dose could upregulate the expression of AR compared with the model group.

The expression of phosphorylated Akt was also detected by Western blot (Fig. 6B). Compared with the control group, the expression of p-Akt (Ser473) in rat testis was dramatically decreased after heat exposure. The expression of p-Akt (Ser473) in the medium-dose WZYZ group was increased significantly compared with the model group. But the differences in other WZYZ groups were not statistically significant. Phosphorylated Akt can can not only regulate the formation, opening and closing of blood-testis barrier (BTB), but also regulate the proliferation, differentiation and spermatogenesis of spermatogonia^62–64^.

Stress damage of testicular tissue caused by HS is also the main cause of spermatogenesis disorder^65^. EPO exerts its action through specific binding to the erythropoietin receptor (EPOR), which is a member of the cytokine receptor superfamily^66^. As shown in Fig. 6C, THS significantly reduced the expression of EPO in testis, but could not affect EPOR. Compared with the model group and even the control group, the expression of EPO and EPOR increased significantly after WZYZ pretreatment. We speculated that EPO may be an important upstream regulator of WZYZ in protecting spermatogenesis induced by acute HS.

### WZYZ inhibited activation of TLR/NFκB signaling pathway by upregulating TAM receptor after THS

P65 is a key molecule of NF-κB family^67^. Activated p65 regulates inflammatory response by regulating gene expression during tissue injury and stress. When a variety of external stimulation signals are transferred into the cytoplasm through TLR, p65 can be transferred into the nucleus by activating the phosphorylation of IκBα, which can bind to the κB motif of its target gene promoter or enhancer, and induce the transcription and expression of related genes^20^. TLR activate specific inflammatory factors and recognize pathogens. MyD88 signal transduction molecule is one of the important adaptors of TLR pathway. TLR4 can sense membrane stimulation and activate downstream molecules through MyD88 signal transduction. Previous studies have shown that TAM receptor can negatively regulate the expression of TLR4 and TLR ligand MyD88^36,68–70^. The results showed that THS induced the activation of IκBα and NF-κB while the mRNA and protein expression of MyD88 changed significantly after THS. There were significant differences in TAM receptor mRNA expression between the groups. WZYZ could restore the down-regulation of TAM receptor mRNA and down regulate the expression of MyD88, p-NF-κB-p65 and p-IκBα induced by THS.

## Discussion

As the occupational THS is becoming increasingly prevalent, in the present study, we examined the protective effects of the traditional Chinese medicine WZYZ against testicular dysfunction in an established rat THS model.

Testicular weight reduction was the most obvious result of the exposure to THS (Fig. 2). Such reduction was in agreement with a previous report that noted a 40% decrease in the weight of adult rat testes in 2 weeks following slightly milder THS than the one used in our study (43 °C, 15 min)^71^. This decrease was attributed to germ cell apoptosis, and we also observed that testicular weight loss was accompanied by spermatogenic cell apoptosis. The TUNEL assay showed that germ cell apoptosis in the model group was significantly higher than that in the control group (Fig. 3). Apoptosis is a programmed cell death process that occurs in many tissues as part of the normal physiological response to injury. However, excessive apoptosis aggravates tissue damage. Notably, germ cell apoptosis in the treatment groups was significantly lower than that in the model group, suggesting that WZYZ protected against THS-induced germ cell apoptosis. The dynamic balance between germ cell proliferation and apoptosis is key to normal spermatogenesis. Ki67 is a marker of cell proliferation^72^, which is mainly expressed in early germ cells, such as spermatogonia and primary spermatocytes. Our previous studies have shown that WZYZ promoted the proliferation of germ cells, so the number of Ki67-positive cells in the spermatogonia of young rats increased after treatment with WZYZ^44^. The results of our current experiments showed that the numbers of Ki67-positive cells in the treatment groups were significantly higher than that in the model group (Fig. 4), which confirmed that WZYZ reversed the inhibition of adult rat germ cell proliferation induced by THS.

Previous studies have reported that the THS-induced spermatogenesis deficit is accompanied by a transient decrease in serum T concentration and increases in FSH and LH levels^73–75^. The decrease in T was attributed to the inhibition of steroidogenesis by THS^76^, which was consistent with our observation that expression levels of T and of its receptor, AR, decreased significantly after THS. We also found that WZYZ treatment restored T and AR expression levels (Figs. 2 and 6). Seminiferous tubules are the site of spermatogenesis. T regulates spermatogenesis only by binding with AR in Sertoli cells. Previous studies have shown that AR-expressed on Leydig cells has limited sensitivity to the level of T. Therefore, the expression of AR (Leydig) did not significantly down regulate with the decrease of T level. However, some studies have reported that the effect of THS on the T content is very small and can be ignored, because the Leydig cells can maintain normal T secretion through a compensatory production mechanism in 2 weeks after the acute THS. FSH secreted by the anterior pituitary is involved in the proliferation and differentiation of testicular spermatogonia, whereas LH stimulates the secretion of T by the Leydig cells. FSH (directly) and LH (indirectly through T and AR) regulate spermatogenesis by stimulating the secretion of cytokines by the SCs^77^. In our experiments, contrary to our expectations, we have not observed the induction of serum FSH and LH levels by THS. The differences between some of the previously published results and our observations can be explained by the different timing of sample collection. Because we sought to evaluate the changes in sex hormone levels in the testis as soon as possible after THS, we did not wait for 1 week or longer, as was done in previous studies^71^, but performed analysis of samples collected 30 min after THS. It is likely that the hypothalamic–pituitary–adrenal axis of rats did not have enough time to respond to changes in T levels induced by acute THS and upregulate FSH and LH levels through a negative feedback mechanism.

Our data showed that the expression of p-Akt in the testis was significantly decreased after THS, whereas the expression of p-Akt was upregulated by the medium-dose WZYZ treatment (Fig. 6). Interestingly, THS inhibited the expression of p-Akt, T, AR, and EPO, whereas WZYZ tended to prevent these changes, promoting cell proliferation and inhibiting cell apoptosis. It has been reported that T activates the Akt signaling via the AR pathway and thereby regulates cell proliferation and apoptosis. In addition, T also mediates the synthesis and secretion of EPO^78^. It has been shown that T injection increased the expression of EPO mRNA in animal testes, and the content of EPO is directly related to the number of red blood cells. Therefore, it has been suggested that the promoting effect of T on the expression of EPO may be one of the reasons for the sex difference in the number of red blood cells. EPO exerts its effects by the interaction with EPOR, which is found on the surface of not only red blood cells, but also of the Leydig cells^79^. EPO has been demonstrated to activate the Akt pathway, protect cell proliferation, and inhibit apoptosis^80^ under various stress states^81–84^. These effects have been confirmed in the studies of the effects of treatment with EPO on testicular torsion and cryptorchidism ischemia–reperfusion^85,86^. EPO could also stimulate the secretion of T^87,88^, which may be related to the activation of PKC, but the specific mechanism of this process remains unclear. The overview of the previously published results and of our present observations suggests that WZYZ regulates the proliferation and apoptosis of testicular cells by activating the Akt signaling pathway through its effects on the levels of T, AR, and EPO, which all may protect against testicular injury induced by THS.

**Figure 7:**
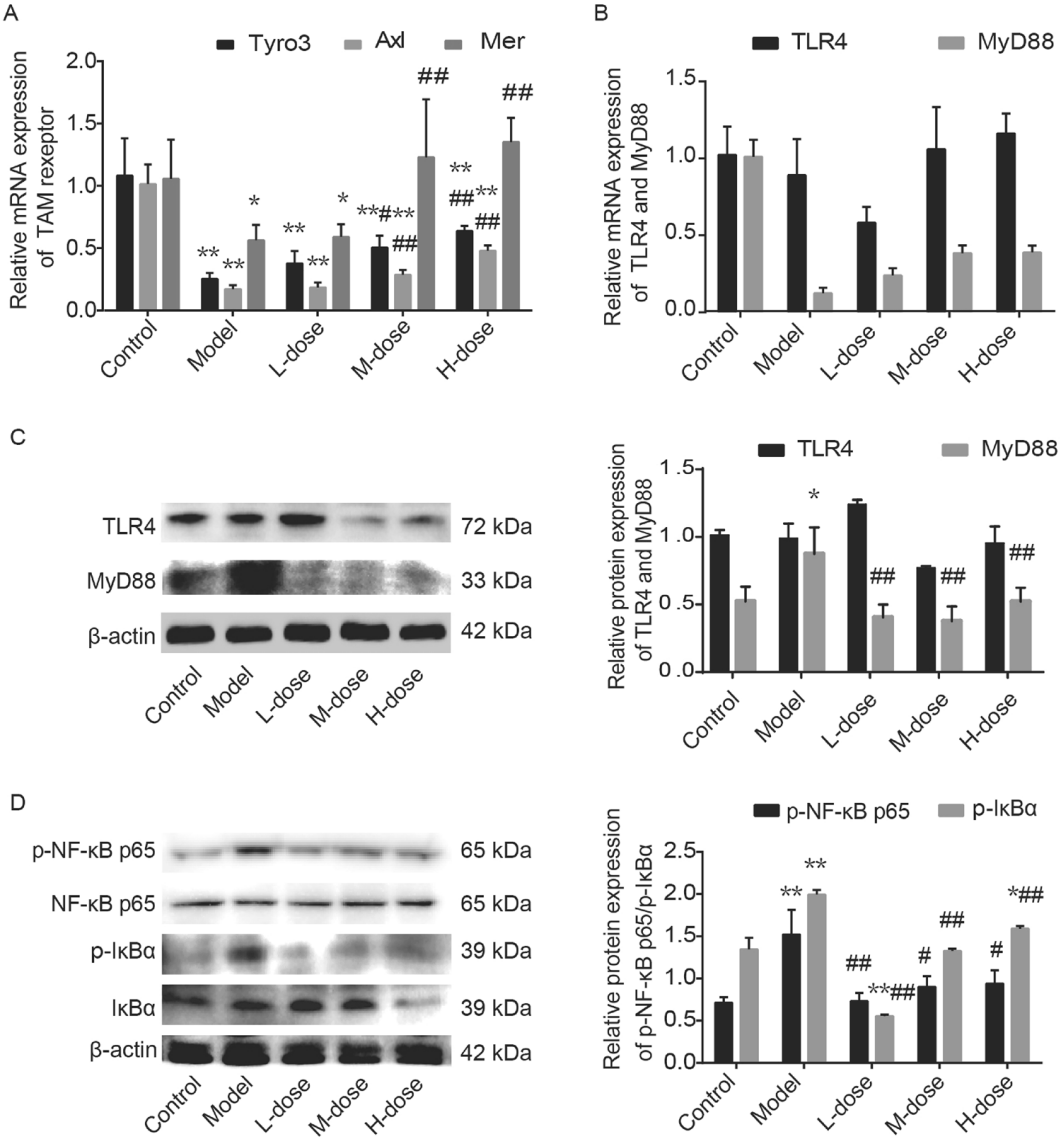
Pretreatment with a gradient concentration of WZYZ upregulated the mRNA expression of TAM receptor and down-regulated the expression of TLR ligands MyD88, phospho-NF-Κb-p65, and phospho-IκBα in acute THS induced rat testis. Analysis the mRNA expression of Tyro3, Alx and Mer (**A**), TLR4 and MyD88 (**B**) in rat testis by RT-PCR. Analysis the expression of TLR4 and, phospho-NF-Κb-p65, NF-Κb-p65, phospho-IκBα, and IκBα (**D**) expression in rat testis by Western blotting. Data are shown as mean ± SEM (n = 3 rat per group). *p < 0.05, **p < 0.01 compared with the control group; ^#^p < 0.05, ^##^p < 0.01 compared with the model group.

THS has been previously shown to activate NF-κB^26^, which is consistent with our results. MyD88 is an important signal transduction molecule in the TLR/NF-κB pathway, which is closely related to the activation of NF-κB. Interestingly, THS inhibited the expression of T, EPO, and TAM receptors (Fig. 7). The latter regulate the inflammatory response by inhibiting the TLR-MyD88/NFκB pathway. In the WZYZ pretreatment group, TAM receptors were up-regulated whereas the expression of MyD88 both at transcriptional and translational level and the protein expression of p-NF-κB-p65 and p- IκBα were downregulated, suggesting that WZYZ targeted TAM receptors and thereby inhibited the Toll-MyD88/NF-κB pathway. The difference of TLR4 expression in WZYZ treatment groups is not statistically significant. However, the pattern of change in the TLR4 expression is consistent with our other results. For the results of testicular weight, TUNEL detection of apoptosis, Ki67 cell proliferation, CK-18, p-Akt, and p-IKB, the optimal effect is found in the medium does group. This phenomenon could be attributed to the multi-target characteristic of WZYZ, a complex compound. Other TLRs, such as TLR2, TLR3, and TLR5^69,89^, could also mediate the activation of the NF-κB signaling pathway. Therefore, THS may activate NF-κB through other TLRx-MyD88 pathways. Although NF-κB was activated in the model group, the expression levels of IL-1 β, IL-2, IL-4, and TNF-α did not change significantly. This result was surprising, but it is consistent with the observations in other studies^71^, which may be attributed to the fact that the selected THS model is relatively moderate compared to those in other studies^9^. Testicular heat stress might be too mild to disturb the systemic immune balance, which prohibited the increase of serum inflammation factors. MyD88 and NF-κB signaling pathway has been proved to be involved in THS induced testicular injury in our study. However, it is worth noting that in addition to the common MyD88-dependent pathway, TLR3 and TLR4 can also use another MyD88-independent signaling pathway, in which the nuclear translocation of transcription factor IFN regulatory factor 3 (IRF-3) activates the expression of typical inflammatory genes, such as interferon and RANTES^90^. So far, it is not clear whether they play a role in THS and how they play these roles. The underlying mechanism require further investigation.

It should be noted that some studies have pointed out that Akt acts as an upstream regulator of NF-κB, and its activation may, in turn, trigger the activation of NF-κB. However, according to our experimental results, the expression level of p-Akt, which is an index of Akt signaling pathway activation, was significantly lower, whereas the expression of p-NF-κB-p65 was significantly higher in the model group than in the control group. This result is consistent with some previous studies^91–93^, An Akt phosphorylation site was found on amino acid residues of IκBα kinase. This suggests that phosphorylation of IκBα (phosphorylation of NF-κB-p65 induced by phosphorylation of IκBα) occurs before phosphorylation of Akt. The published data and our present results, therefore, indicate that the Akt and NF-κB signaling pathways play parallel roles in mediating the consequences of THS.

Compared with non-immune privileged tissues, the testis has a greater capability to inhibit inflammatory reactions, which is reflected in the fact that a variety of cells, such as SCs and stromal cells, secrete various immunosuppressive factors, including T, express TAM receptors, and have the blood-testis barrier (BTB), the most important physical structure that confers immune privilege. The BTB is formed by the SCs through the tight junctions between cells. In addition to regulating permeability, providing nutrients and hormones for germ cells^45^, preventing cytotoxic substances from entering seminiferous tubules, and helping spermatogenic cells migrate^94^, the BTB protects germ cells by isolating them from the immune system^95^. Previous studies have shown that THS interferes with the BTB assembly^96^, which is consistent with our electron microscopy observations (Fig. 5). The tight junctions of the testicular mesenchymal stem cells became loose after acute THS, and the structure of the testicular mesenchymal stem cells was closer to that of the normal group in the animals treated by WZYZ. In the model group and some WZYZ treated groups, vacuoles are found in the seminiferous tubules. Vacuolization of seminiferous tubules might be due to radiation^97,98^, heavy metal poisoning^99,100^ as well as heat stress^101–103^. It is generally believed that vacuolization might be related to spermatogenic disorder. Whether vacuolization is positively correlated with the severity of spermatogenic disorder is still inconclusive. The differentiated SCs form the BTB^55^ and create a suitable environment for spermatogenic cell proliferation. Our previous studies showed that the expression of CK-18 increased in undifferentiated SCs, but decreased or even disappeared after SCs had become fully differentiated^44^. In this study, CK18 expression measurements showed that THS likely induced the dedifferentiation of SCs, whereas WZYZ treatment inhibited that SC dedifferentiation trend (Fig. 5). Here, we need to emphasize the important role of the AR, which is mainly expressed in SCs before the formation of BTB. The latter circumstance suggests that AR plays a key regulatory role in the differentiation of SCs and in the formation of BTB^55^. SCs lacking AR could not form tight junctions or exhibit phagocytic function^104^, and failed to provide proper nutrition and protection for germ cells^105^, which led to spermatocyte development stagnation, premature separation of immature sperm cells from SCs, and failure of mature sperm release from the SCs. Therefore, the upregulation of AR expression by WZYZ may be the key mechanism whereby this preparation could inhibit THS-induced dedifferentiation of SCs and promote repair of the BTB structure.

**Figure 8:**
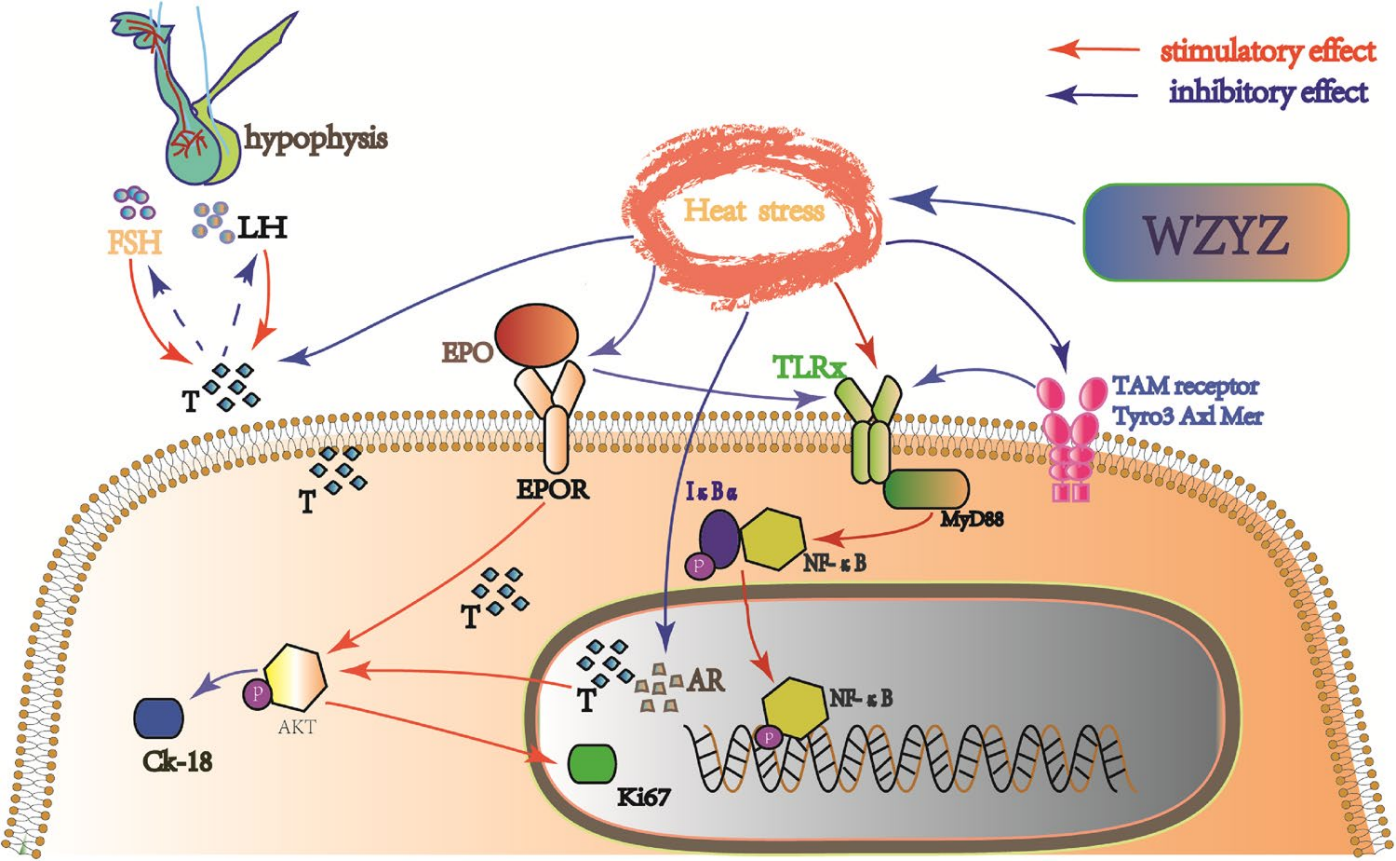
Proposed mechanisms of Wuzi-Yanzong prescription alleviates Spermatogenesis Disorder induced by acute THS.

It has been increasingly recognized that the inherent functions of the testis, such as spermatogenesis, sex hormone synthesis, and immune regulation, are connected by an interactive and complex network. On the one hand, under normal circumstances, the immune response is a local tissue defensive reaction that timely removes the fragments of apoptotic cells and restricts the extent of the damage. On the other hand, if the immune system is dysregulated, the excessive levels of inflammatory factors stimulate immune cells to release large amounts of inflammatory mediators. Such outbreak of inflammation negatively affects spermatogenesis. The need for a delicate balance between the two modes of immune system functioning is reflected in our research. Here, we preliminarily conceive a mechanism of Wuzi Yanzong prescription to alleviate spermatogenesis disorder caused by acute THS, as shown in Fig. 8.

## Conclusion

This study demonstrates that WZYZ ameliorated the spermatogenesis deficit induced by THS via modulation of the Akt and NF-κB signaling pathways. This modulation was, in turn achieved by the WZYZ effects on the levels of T, AR, EPO, and TAM receptors. Our results show that WZYZ induces resistance to THS, which may provide a new therapeutic strategy for the treatment of heat stress-related male infertility.

## Supplementary Information

Supplementary Information.
