# Supplementary material for: Wuzi-Yanzong prescription alleviates spermatogenesis disorder induced by heat stress dependent on Akt, NF-κB signaling pathway
Source: Sci Rep. 2021 Sep 22;11:18824. doi: 10.1038/s41598-021-98036-2 (PMC8458393; doi:10.1038/s41598-021-98036-2)
Supplement: Supplementary file 1 — Supplementary Information. [file 41598_2021_98036_MOESM1_ESM.pdf]

Supplementary material for the manuscript entitled "Wuzi-Yanzong prescription alleviates Spermatogenesis Disorder induced by Heat stress dependent on Akt, NF-κB signaling pathway" (ID: 452f94b8-d44b-47be-af85-05303dd2884e).

We admit that the image of the original blots was closely cropped. We have made the following explanation in the method of the manuscript:
“According to the molecular weight of the target protein, the important stripes were cropped on the gel, and the appropriate bandwidth above and below was reserved. Due to the molecular weight of some target proteins is close, we have retained as much information as possible.”
Due to the different contrast between different target protein bands and the background, the membrane edges of some images are not visible after auto exposure. In the following picture, for example, membrane edge of the upper bolt can be showed clearly after exposure, while the lower bolt can't show the membrane edge.

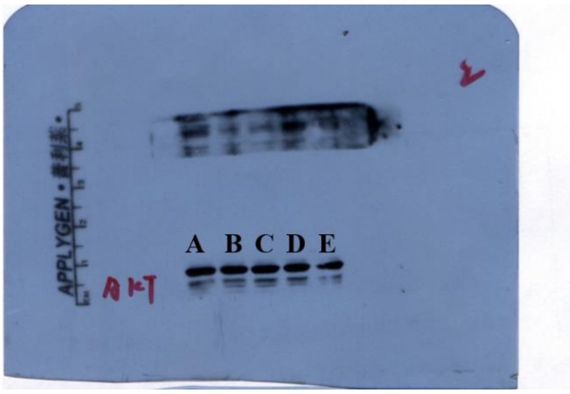

We have selected clear and complete bolts in the original image. All the figures have been corrected and are consistent with the bolts provided in the submitted manuscript. The selected bolts with multiple bolts in the original image have been marked with red box.
We want to provide images that retain the original information to the maximum extent possible. We will pay more attention to these specifications in future research and we appreciate and thank you for your rigorous and meticulous work.

1.Fig.5B CK-18

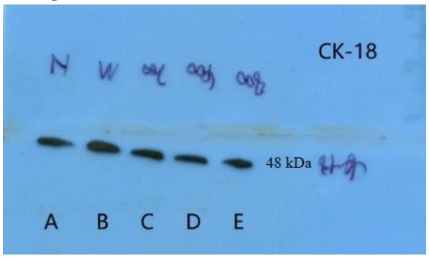

β-actin

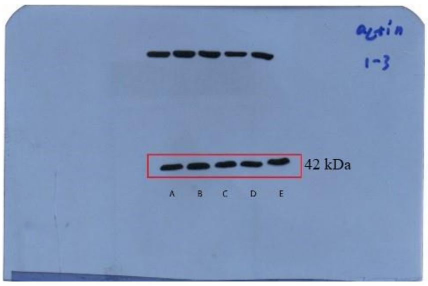

2. *Fig.6A AR*

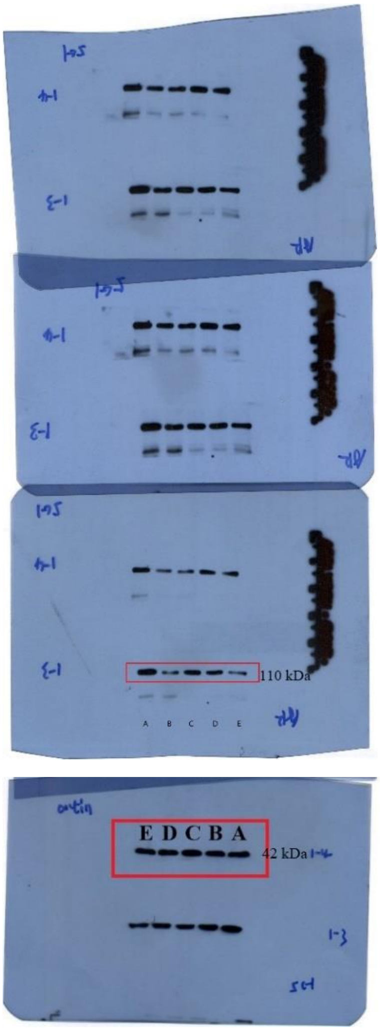

3. *Fig.6B Akt、P-Akt*

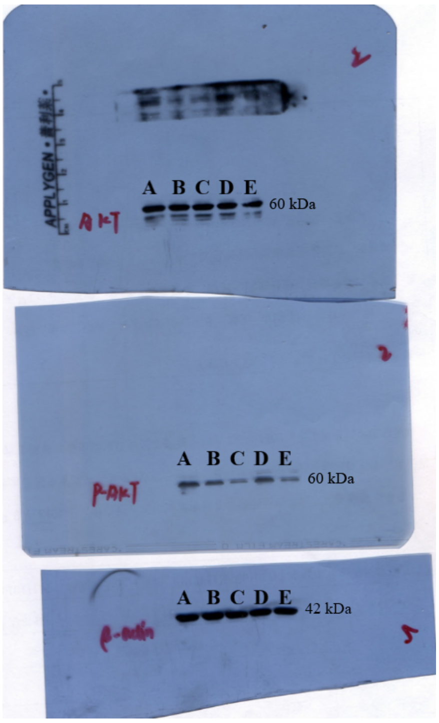

4. Fig.6C EPO、EPOR

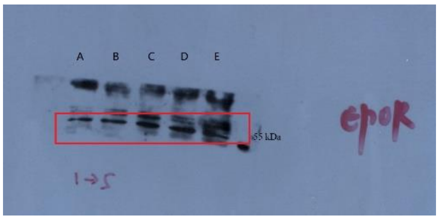

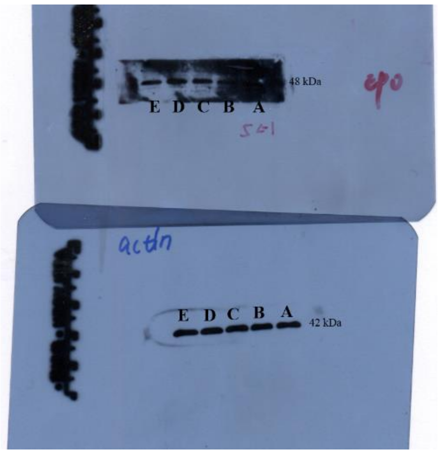

5. Fig.7C TLR4、MyD88

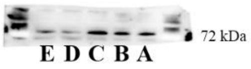

TLR4

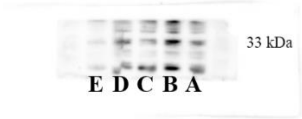

MyD88

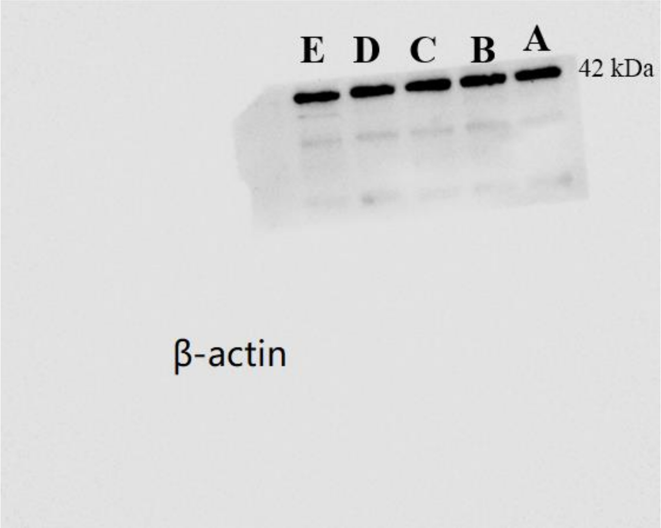

6.Fig.7D IκBα、p-IκBα、NF-κB-p65、p-NF-κB-p65

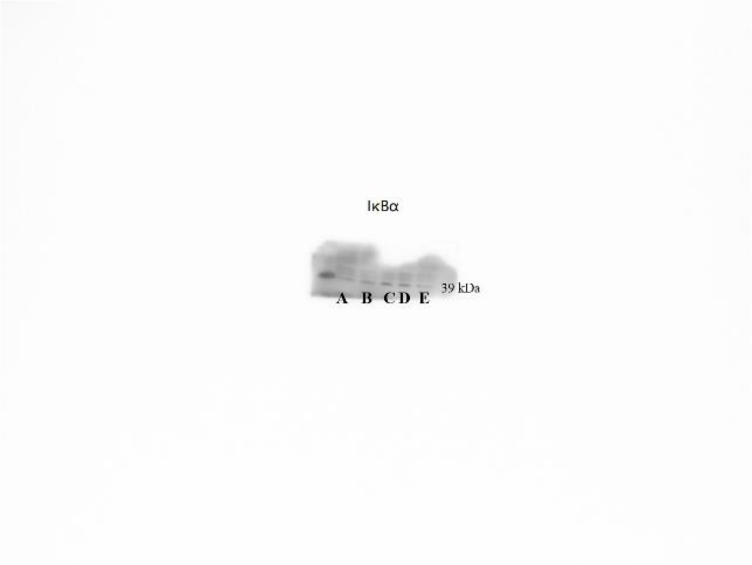

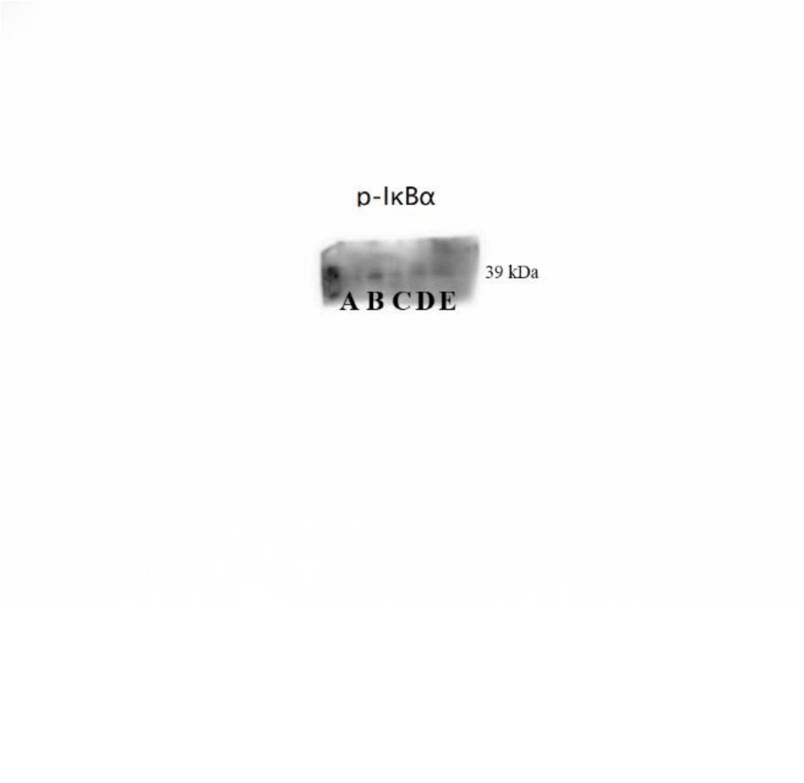

NF-κB-p65

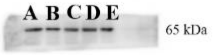

p-NF-κB-p65

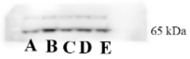

β-actin

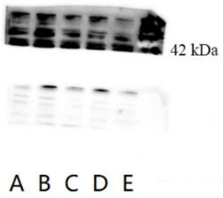

Gray value of statistical data

p-IκBα

	lane1	lane2	lane3
A	12732.03	11771.86	12771.86
B	25114.68	22753.97	25135.97
C	6825.711	5885.761	6679.589
D	16520.51	15917.1	16869.22
E	16419.79	14502.77	15877.97

IκBα

	lane1	lane2	lane3
A	7956.401	10496.02	9742.874
B	12431.07	12083.58	12107
C	11592.32	11181.9	12095.51
D	12892.31	11617.46	12694.87
E	9943.56	9259.317	10229.39

p-IκBα/IκBα

	1	2	3
A	1.600225	1.121554	1.310893
B	2.020316	1.883049	2.076153
C	0.588813	0.526365	0.552237
D	1.281424	1.370101	1.328821
E	1.651299	1.56629	1.552191

	β-actin			***tlr4***			***myd88***		
A	19.94982	23.08468	23.36913	21.08918	22.992	22.99389	18.56874	19.3185	16.21853
B	25.69694	26.0511	21.51743	24.17687	23.64684	23.97525	44.20949	42.33944	49.12378
C	24.02596	22.19462	21.83543	29.48086	28.37234	26.41836	13.55226	14.07028	15.70628
D	19.48969	18.27907	21.84065	14.93111	13.78896	17.14269	19.65907	20.00007	15.08173
E	10.8376	10.39052	11.43736	10.32198	11.19986	9.469818	4.010449	4.271714	3.869686

	tlr4/β-actin			***myd88/β-actin***		
A	1.057111	0.995985	0.983943	0.930772	0.836854	0.694015
B	0.940846	0.90771	1.114224	1.720419	1.625246	2.282976
C	1.227042	1.278343	1.209885	0.564067	0.63395	0.719302
D	0.766103	0.754358	0.784898	1.008691	1.094151	0.690535
E	0.952424	1.077892	0.827973	0.37005	0.411116	0.338337

Repeat experiment on TLR4 MyD88 p-ikb

TLR4

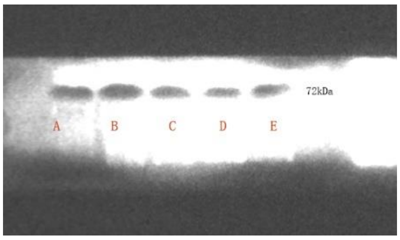

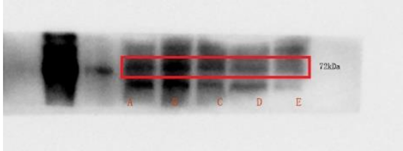

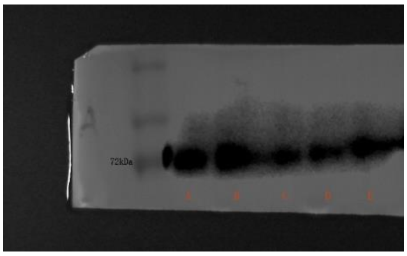

MYD88

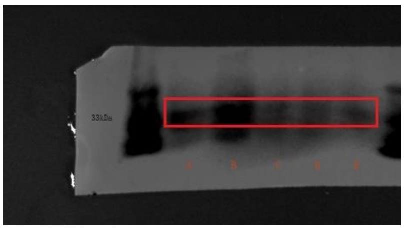

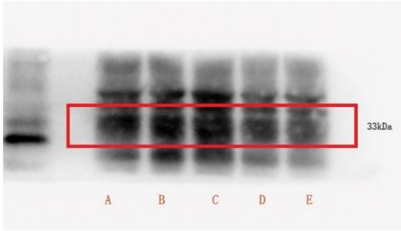

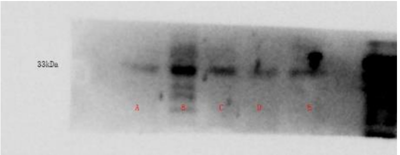

p-ikb

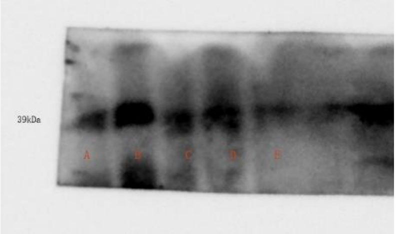

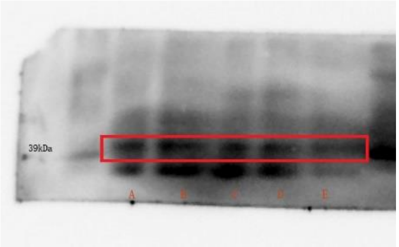
39kDa

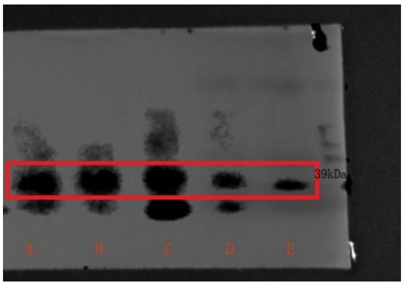
39kDa
A B C D E

Ikb

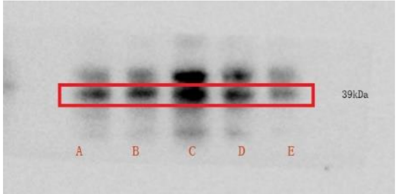
39kDa
A B C D E

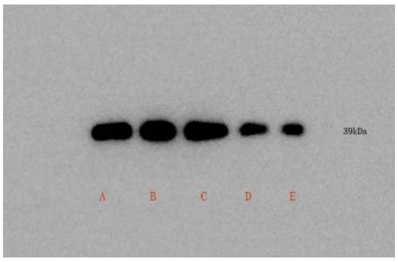
39kDa
A B C D E

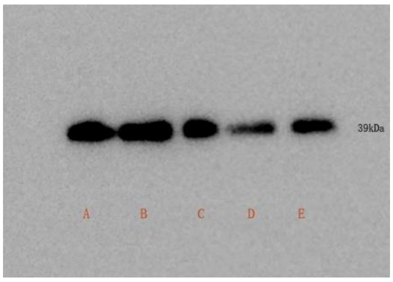
39kDa
A B C D E

β-actin

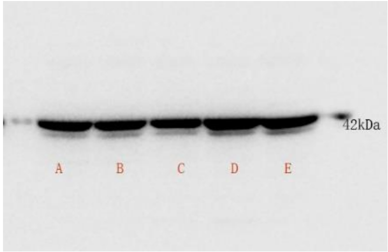
42kDa
A B C D E

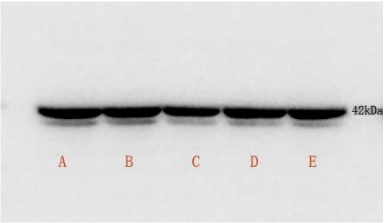
42kDa
A
B
C
D
E
